# Long QT interval syndrome type 2 caused by a new missense mutation of *KCNH2* gene: A case report

**DOI:** 10.1097/MD.0000000000046991

**Published:** 2026-01-16

**Authors:** Yixiang Ma, Li Wang, Shuqi Wu, Chang Peng

**Affiliations:** a Department of Pediatrics, Guizhou Children’s Hospital, Affiliated Hospital of Zunyi Medical University, Zunyi, Guizhou Province, China.

**Keywords:** child, *KCNH2* gene, long QT interval syndrome, mutation

## Abstract

**Rationale:**

Long QT syndrome (LQTS) constitutes an inherited cardiac. Studies indicate that untreated LQTS carries a high mortality rate, and up to 20% of sudden infant death syndrome cases are associated with this condition. With active therapeutic intervention, the disease mortality rate can be reduced to 1%.

**Patient concerns:**

We report a 10-year-old male patient who has a sudden loss of consciousness. The electrocardiogram showed the QT interval was prolonged, which means LQTS. The results of genetic testing found heterozygous mutations, c.1943 G > C, p.Gly 648 Ala in exon 7 of the *KCNH2* gene, it was missing mutations and had not been reported before.

**Diagnoses:**

This patient was diagnosed with LQT interval syndrome based on the medical history, physical examination, electrocardiogram, and genetic testing.

**Interventions:**

The patient was hospitalized for a duration of one week and received treatment comprising an oral β-blocker (propranolol: 2–4 mg·kg^−1^·d^−1^), potassium supplementation, and symptomatic management. No further syncopal episodes occurred. In the following year, the patient’s QT interval on the electrocardiogram gradually returned to the normal range. The long-term prognosis requires continued monitoring.

**Outcomes:**

We provided a standard treatment plan for the child patient. So far, the child has not experienced fainting again, reducing the possibility of sudden death.

**Lessons:**

We reported a new missense gene mutation that caused LQT2 syndrome. This case underscores the critical importance of early identification, diagnosis, and treatment of LQTS to significantly mitigate the risk of sudden death in children.

## 1. Case report

The patient was a 10-year-old male admitted to the Department of Pediatrics, Affiliated Hospital of Zunyi Medical University, presenting with sudden loss of consciousness (LOC). The child experienced an abrupt LOC without identifiable precipitants, persisting for approximately thirty minutes. Preceding the onset, there was an absence of fever, cough, dyspnea, chest tightness, palpitation, precordial pain, respiratory distress facial cyanosis, irritability, headache, dizziness, emesis, diarrhea, convulsion, or urinary or fecal incontinence. No prior history of similar episodes was documented. The patient denied any history of illicit substance use, recent infection, or cranial trauma. Family history was negative for similar medical conditions and for sudden cardiac death.

Physical examination revealed a temperature was 36.8 ℃, pulse of 54 beats per minute, respiration rate of 21 breaths per minute, the blood pressure of 104/65 mm Hg, and weight of 32 kg. The patient was conscious and oriented. Examination of the face and lips exhibited no cyanotic or pallor, and no enlarged lymph nodes were palpable in the cervical region. Pulmonary auscultation revealed no abnormalities bilaterally. The cardiac rate was 54 beats per minute with an irregular rhythm. Cardiac sound were strong; percussion revealed no enlargement of the cardiac borders, and auscultation over the valve areas detected no pathological murmurs. Abdominal and neurological examinations were unremarkable, and no edema was present in the lower extremities.

**Figure 1.**
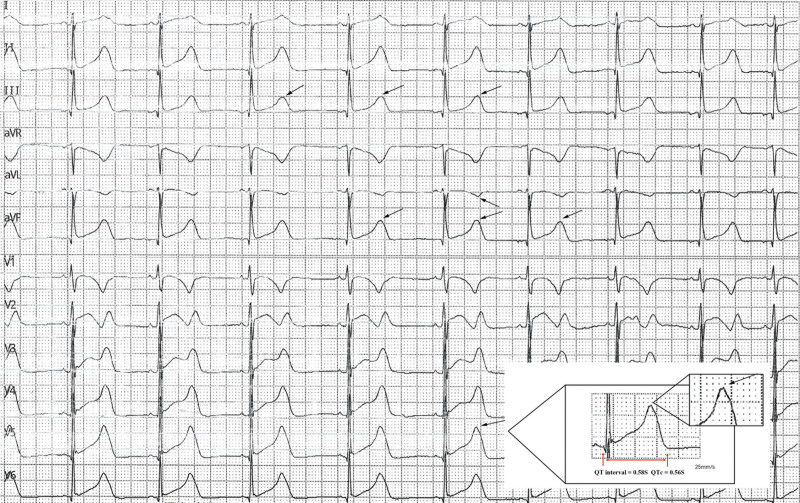
The ECG data of the LQTS patient. QT interval = 0.58 s, QTc = 0.56 s, a square represents a time interval = 0.2 s. T wave can see the notch in III, aVL, aVF, and V5 lead (black arrows). ECG = electrocardiogram, LQTS = long QT intervals syndrome.

**Figure 2.**
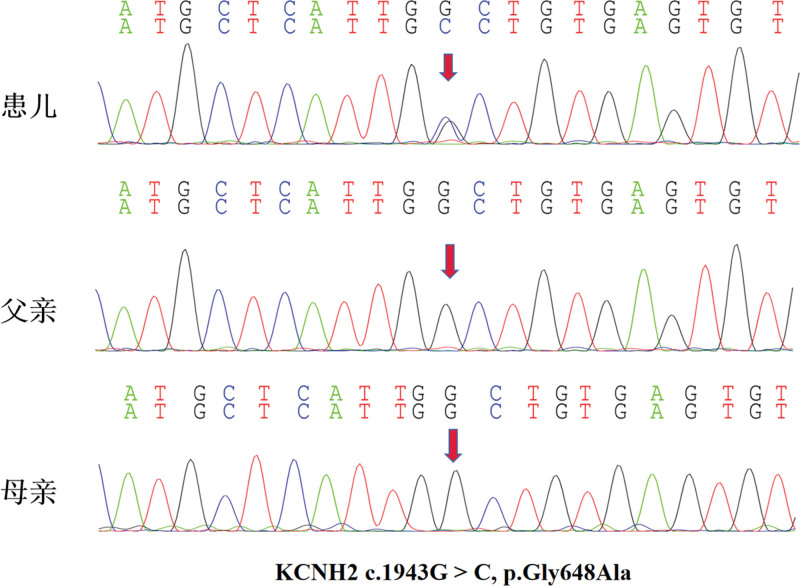
Gene sequencing map (transcript version number: NM_000238).

The patient had previously enjoyed good health without known medical conditions. His father possesses a diagnosis of dilated cardiomyopathy, exhibiting a left ventricular ejection fraction (EF%) of 35% and a normal electrocardiogram (ECG). The patient’s mother is in good healthy. Laboratory investigations, including complete blood count, serum electrolytes, liver function tests, thyroid function tests, renal function tests, cranial magnetic resonance imaging, echocardiogram, and electroencephalogram (EEG), were all within normal limits. The ECG demonstrated sinus bradycardia with sinus arrhythmia, a prolonged QT interval (0.58 seconds), and elevated potassium levels, as illustrated in Figure 1. Based upon the patient’s history and auxiliary investigations, a diagnosis of long QT syndrome (LQTS) was established. Following the provision of informed consent by the parents, 2 mL of intravenous blood was collected from the child and both parents for genetic analysis. Whole exome sequencing targeting arrhythmia-associated genes was performed on the proband’s sample utilizing next-generation sequencing technology (conducted by Beijing Kangxu Medical Laboratory). Identified mutation sites in the proband were subsequently verified in the parental samples via Sanger sequencing. The genetic analysis identified a heterozygous missense mutation in exon7 of the *KCNH2* gene, designated c.1954G > C, p.Gly648 Ala. This variant was classified as pathogenic according to the guidelines established by the American College of Medical Genetics and Genomics (ACMG). As depicted in Figure 2 pathogenicity prediction software (SIFT, Polyphen2, MutationTaster) Assessed the *KCNH2* gene mutation c.1943G > C, p.Gly648Ala. MutationTaster predicted this variant as likely pathogenic, Polyphen2 predicted it as probably damaging, and SIFT predicted it as tolerated.

## 2. Discussion

### 2.1. Hospital course

Following admission, the patient underwent comprehensive diagnostic investigations, including complete blood count, electrolyte panel, hepatic function tests, renal function tests, myocardial enzyme assays, thyroid function studies, echocardiography, electroencephalography, and electrocardiography. All findings were within nomal limits. Based on the clinical history and electrocardiographic presentation, a diagnosis of LQTS was established. Subsequent genetic testing was performed. The patient was hospitalized for a duration of 1 week and received treatment comprisiong an oral β-blocker (propranolol: 2–4 mg·kg^−1^·d^−1^), potassium supplementation and symptomatic management. No further syncopal episodes occurred. In the following year, the patient’s QT interval on the electrocardiogram gradually returned to the normal range. The long-term prognosis requires continued monitoring.

### 2.2. Discussion of case and literature

LQTS was first described in the 1950s and initially appeared in a deaf family (namely Jervell and Lange Nielsen syndrome).^[1]^ A few years later in the early 1960s, patients with similar ECG abnormalities without deafness (i.e., Romano–Ward syndrome) were described.^[2]^ Its typical features are prolongation of the QT interval on ECG and occurrence of a sudden LOC, cardiac arrest, and sudden cardiac death, which mainly caused by emotional changes or physical stress.^[3–5]^ Some study showed that untreated LQTS has a high mortality rate, and up to 20% of sudden infant death syndrome is associated with this disease.^[6,7]^

LQTS constitutes an autosomal dominant cardiac ion channelopahty inherited within families. Nevertheless, sporadic LQTS cases wherein parents exhibit no pathogenic genetic variants.^[8]^ The pathogenesis of LQTS is primarily arises from mutations affecting genes encoding ion channels subunits or proteins that indirectly regulate ion channel function.^[9]^ Diagnosis relies up on clinical manifestations, electrocardiographic evidence of prolonged QT intervals, and genetic testing. When the QT interval exceeds 0.5 seconds, the risk of cardiac arrest and sudden cardiac death is significantly increased.^[10]^ Consequently, upon electrocardiographic indication of QT interval prolongation genetic testing should be performed expeditiously. Following diagnosis confirmation, early intervention measures must be implemented to prevent sudden cardiac death.

In addition to the long QT interval syndrome caused by genetic factors, drugs, electrolyte imbalances and hyperthyroidism can also lead to prolonged QT intervals.^[11]^ Drugs such as antiarrhythmic drugs, antibiotics, antipsychotics, antihistamines, antiemetics, etc, all have the risk of causing prolonged QT intervals.^[12,13]^ Electrolyte imbalances such as hypokalemia and hypomagnesemia are also recognized as risk factors. When both hypokalemia and hypomagnesemia occur simultaneously, their combined effect on the QT interval can be synergistic.^[11]^ The diseases that cause hyperthyroidism can also lead to an elongated QT interval.^[14]^ Therefore, when diagnosing hereditary long QT interval syndrome, these factors need to be excluded.

The most common types of LQTS are LQT1 (40%–55%), LQT2 (30%–45%), and LQT3 (5%–10%). LQT is caused by the loss of function of *KCNQ1* channel encoding I_Ks_ (slow activation of delayed rectifier potassium channels), LQT2 is caused by the loss of function of *KCNH2* channel encoding I (fast activation of delayed Kr rectifier potassium channels), and LQT3 is caused by the rapid activation of cardiac sodium channels caused by mutations in the *SCN5Aa* gene.^[15,16]^ QT interval duration can not distinguish the types of LQTS, while T wave morphological changes can be used to describe different phenotypes of LQTS. For example, LQT1 eledtrocardiograns typically have broad T waves and LQT2 has a visible notch in the T waves (bimodal T waves),^[15]^ and LQT3 shows a highly sharp T wave.^[17]^

LQT2 is the most common type of LQTS and is commonly caused by heterozygous mutations in the *KCNH2* gene on chromosome 7q36. *KCNH2* encodes the alpha subunit of K.V11.1 channel, which is the basic action potential of rapid activation and delayed rectified K^+^ current (Ⅰkr) in cardiac phase 2 and 3, and thus plays an important role in cardiac repolarization.^[18,19]^ The possible mechanisms of *KCNH2* gene mutation leading to LQT2 are as follows: class Ⅰ mutation disrupts the synthesis of *KCNH2* encoded Kv11.1 subunit; class Ⅱ mutations disrupt Kv11.1 channel protein intracellular trafficking or translocation to the cell membrane; class Ⅲ mutations disrupt IKr channel; class IV mutations disrupt the permeability/selectivity of channel IKr. The mutant *KCNH2* gene causes IKr channel dysfunction through different mechanisms (mainly class Ⅰ and class Ⅱ) prolongs the action potential time of cardiomyocytes, and leads to delayed ventricular repolarization, and so the ECG shows abnormally prolonged QT interval, and prolongation of QT interval increases the risk of malignant arrhythmias such as fatal torsades de pointes (TdP) and ventricular fibrillation.^[20,21]^ Studies have shown that more than half of the *KCNH2* gene mutations are missense mutations, and functional studies have shown that about 90% of these mutations will destroy the *KCNH2* encoded Kv11.1 channel protein and affect the cell membrane to intracellular transport, so missense mutations are closely related to the pathogenesis of LQTS.^[22,23]^

This patient exhibited LOC. The electrocardiogram revealed a QT interval exceeding 500 ms, accompanied by a Schwartz score of 4 points. Secondary long QT syndrome was excluded through laboratory investigations, permitting a diagnosis of LQTS according to established guidelines.^[24]^ For patients presenting with a Schwartz score exceeding 3.5 points, current consensus strongly recommends genetic testing, such testing not only facilitates the diagnosis of LQTS but also enables the identification of specific genotypes and the determination of whether the patient’s family members harbor pathogenic mutations.^[25,26]^ Genetic analysis identified a heterozygous mutation (c.1943G > Cp.Gly648Ala) in exon 7 of the *KCNH2* gene in this LQTS patient. This specific mutation was previously unreported within the HGMD pro, Pubmed and Clinvar databases. Pathogenicity prediction utilizing SIFT, Polyphen2, and MutationTaster, indicated this novel mutation to be highly pathogenic (detailed results presented in Table 1). Consequently, the discovery of this novel *KCNH2* gene mutation site will further enrich the LQTS gene database and provide a new reference for the clinical diagnosis of this disease. However, validation of this mutation site’s cansative role in LQTS necessitates further investigation through animal models of *KCNH2* gene mutation.

**Table 1:** Prediction of protein functional damage in *KCNH2* gene mutation.

Soft name	SIFT	Polyphen2	MutationTaster
Mutation site	c.1943G > C, p.Gly 648 Ala) in exon 7		
Result	Tolerated	Probably damaging	Disease causing
Score	0.04	1	1

In LQT2, a sudden LOC is usually the first symptom, and the risk of sudden cardiac death is very high.^[27]^ Therefore, early genetic testing can provide an important basis for diagnosis, treatment and prognosis. At present, there is no radical treatment for LQTS. *KCNH2* is associated with potassium channel function, so patients with LQT2 are often accompanied by hypokalemia. If potassium concentration cannot be recovered by food and/or oral potassium supplement drugs, drugs that reduce potassium excretion (such as spironolactone) can be considered. However, some studies have shown that for LQTS patients with normal serum potassium concentration, increasing serum potassium concentration can not shorten the QT interval and about 25% of patients have side effects.^[28]^ The trigger factors of arrhythmia events are usually related to acute wake-up, such as quiet rest and a wake-up alarm clock during sleep. It should be considered to keep the telephone and alarm clock away from the child’s resting area. When waking up the child in the morning it should be whispered to avoid loud shouting to stimulate the child to induce the attack of the disease.^[29]^ β-blockers are a mainstay of medication for the prevention of the disease. However, there is still a risk of cardiac arrest and LOC after β-blocker administration. In recent years, defibrillators have been implanted to prevent cardiac arrest and LOC.^[30]^ His child was given β-blocker (propranolol) and oral potassium supplementation for preventive treatment, and no LOC or cardiac arrest occurred. He is still being followed up, but the long-term efficacy and prognosis are subject to further observation.

## 3. Final diagnosis

Based on the patient’s medical history, Schwartz score,^[9]^ ECG findings and genetic testing results, the patient was clinically diagnosed with LQT2.

## 4. Limitations

We identified a novel missense gene mutation resulting in long QT syndrome type 2 (LQT2). Congenital long QT syndrome typically possesses a genetic etiology. However, this identical missense mutation was not detected in the proband’s parents. In subsequent investigations, we will employ gene-knockout animal models to further elucidate and validate the mechanistic relationship between this mutated gene and long QT syndrome.

## 5. Conclusion

LQTS is a disease in which the ventricular repolarization is delayed and tip torsional chamber velocities are frequently seen. LQTS is one of the most common causes of sudden cardiac death in infants and young children, as LQTS is prone to torsional ventricular tachycardia and ventricular fibrillation. Long QT syndrome significantly reduces the risk of sudden death following standard treatment. Therefore, early detection, diagnosis, and treatment of this condition are of critical importance for pediatric patients. Diagnosis and type of LQTS in children is mainly based on ECG and genetic testing.

## Author contributions

**Conceptualization:** Chang Peng.

**Data curation:** Yixiang Ma, Li Wang.

**Formal analysis:** Yixiang Ma.

**Investigation:** Shuqi Wu.

**Methodology:** Li Wang.

**Project administration:** Chang Peng.

**Validation:** Shuqi Wu.

**Writing – original draft:** Yixiang Ma.

**Writing – review & editing:** Chang Peng.
